# Eosinophilic Esophagitis in Children from Western Saudi Arabia: Relative Frequency, Clinical, Pathological, Endoscopic, and Immunological Study

**DOI:** 10.1155/2012/328253

**Published:** 2012-12-04

**Authors:** Omar I. Saadah, Abdullah J. Aburiziza, Rafat I. Abu Shakra

**Affiliations:** ^1^Division of Pediatric Gastroenterology/Department of Pediatrics, Faculty of Medicine, King Abdulaziz University, P.O. Box 80215, Jeddah 21589, Saudi Arabia; ^2^Division of Pediatric Allergy Immunology/Department of Pediatric, Faculty of Medicine, Umm Al Qura University, P.O. Box 16097, Makkah 21955, Saudi Arabia; ^3^Department of Pathology and Laboratory Medicine, International Medical Center, P.O. Box 2172, Jeddah 21451, Saudi Arabia

## Abstract

*Background and Purpose*. Eosinophilic esophagitis (EE) is an evolving allergic disease with an accelerated incidence. The purpose of this study was to delineate the relative frequency and clinicopathological characteristics of EE in children from western Saudi Arabia. *Methods*. Children with EE were studied retrospectively between October 2002 and December 2011 at King Abdulaziz University Hospital and International Medical Center. *Results*. The relative frequency of EE was 0.85% of 2127 upper gastrointestinal endoscopies performed during the study period. Eighteen patients were identified with EE. The median age was 8.6 years (range, 1.5–18 years). Thirteen (72.2%) were males. Dysphagia and vomiting were the most common symptoms. Ten (55.6%) children had history of atopy. Testing for food allergy by skin prick test was positive in 11 (61.1%). The most common endoscopic abnormalities were mucosal longitudinal furrow and loss of vascular pattern followed by patchy specks and strictures. The histopathological findings included increased intraepithelial eosinophils, eosinophilic degranulation, lamina propria fibrosis, and eosinophilic microabscesses. Treatment was initiated by swallowed topical corticosteroids in 12 (66.7%) and oral prednisolone in 6 (33%) patients, followed by low dose of topical corticosteroids and dietary elimination. *Conclusions*. Eosinophilic esophagitis is an uncommon but evolving problem. A high index of suspicion is required for early identifications and intervention to avoid possible complications.

## 1. Introduction

Eosinophilic esophagitis (EE) is defined as a chronic immune-mediated disorder with symptoms of esophageal dysfunction and an esophageal mucosal eosinophilic infiltrate [1]. The prevalence of this clinicopathological condition is increasing in both children and adults which results in significant morbidity [2, 3]. The accelerated incidence of EE reflects a true increase and not merely the result of greater awareness by the physicians [4]. The diagnosis of EE is established if histology demonstrates more than 15 epithelial eosinophils per high-power field (hpf) isolated to the esophageal mucosa with associated upper gastrointestinal symptoms [5]. Alternative causes of esophageal eosinophilia must be ruled out before EE can be diagnosed [6, 7]. Younger children commonly present with abdominal pain, vomiting and feeding aversion, while older children and adolescents have symptoms of heartburn, dysphagia, and acute food impaction similar to adults [8, 9]. There is a frequent association with allergic diseases such as childhood bronchial asthma, allergic rhinitis, atopic dermatitis, and food allergy [8, 10]. The mainstay of EE therapy for children is swallowed topical steroid preparation, with acid suppression and dietary manipulation being useful in selected cases [6]. The reported occurrence of EE in children from Saudi Arabia was limited to only two reports [11, 12].

The aim of this study was to describe the relative frequency and clinical manifestations of EE in a group of children from the western region of Saudi Arabia in order to increase the awareness of the pediatricians and the family physicians to the existence of such disease entity.

## 2. Patients and Methods

### 2.1. Clinical Study

This retrospective and observational study included all children and adolescents less than 18 years of age seen at the pediatric gastroenterology service at King Abdulaziz University and International Medical Center in the period between October 2002 and December 2011 with clinical diagnosis of EE. Patients were identified through searching the medical records of each hospital and the endoscopy database. Data were retrieved from the patient's medical files including demographic data, clinical presentations, anthropometric measurements, laboratory investigations, endoscopic findings, and treatment prescribed. The *z* scores for weight and height were calculated using anthropometric software (Epi-Info, Centers for Disease Control and Prevention, Atlanta, GA, USA). All patients underwent esophagogastroduodenoscopy using Olympus Pediatric Video Gastroscope under either conscious sedation using intravenous midazolam or general anesthesia. The diagnosis was suspected from the clinical presentation and endoscopic appearance and confirmed by histopathological examination of biopsy specimens obtained from the esophagus at different levels (proximal and distal) under direct visualization. Tissue samples were also obtained from the gastric mucosa and duodenum to exclude eosinophilic gastroenteritis.

This study was approved by the Bioethical and Research Committee of Faculty of Medicine at King Abdulaziz University and the International Medical Center Institutional Review Board. The study was conducted according to the principles of Helsinki Declaration.

### 2.2. Histopathological Examination

Esophageal biopsies were initially fixed in 10% NBF from 18 to 24 hours and embedded in paraffin. Sections of 3–5 micron thick were made and stained with Hematoxylin and Eosin (H&E). The sections were reviewed by a certified single pathologist (RA). The eosinophilic count was assessed by counting eosinophils in up to 5 hps with the highest intraepithelial eosinophils count. Only eosinophils which display both a dark cluster of eosinophilic granules and nucleus with one or two lobes were counted. The other histopathological features that were assessed are eosinophilic microabscesses (defined by aggregates of >4 eosinophils), presence or absence of intercellular edema, basal cell hyperplasia more than 20% of the epithelial thickness, elongation of lamina propria papillae to more than two thirds of the epithelial height, presence of eosinophils in lamina propria, assessment of lamina propria fibrosis, eosinophilic degranulation, neutrophilic infiltration, and presence or absence of ulceration. EE was diagnosed with intraepithelial eosinophils more than 15 in more than 2 hpfs or more than 25 in any single hpf [13]. Follow-up biopsies after taking treatment for EE if available were reviewed using the same criteria to assess all previously mentioned histopathological features.

### 2.3. Diagnosis of EE

The final diagnosis of EE in a child with upper gastrointestinal symptoms was made according to the following criteria [1]: (1) evidence of esophageal tissue eosinophilic infiltration as described above; (2) exclusion of gastroesophageal reflux disease (GERD) by either 24-hour pH study or the demonstration of minimal or no response to treatment with proton pump inhibitors; (3) exclusion of other local and systemic causes of gastrointestinal eosinophilia such as eosinophilic gastroenteritis, inflammatory bowel disease, celiac disease, parasitic infection, or systemic eosinophilic syndrome; (4) demonstration of clinical response to treatment directed at EE.

### 2.4. Extended Esophageal pH Study

Extended esophageal pH study was performed by one of the authors (OIS) using a 2.1 mm pH catheter with two antimony electrodes (Medtronic Synectics, Shoreline, MN). The pH electrodes were calibrated in pH 7.0 and 1.0 buffer solutions (Medtronic Synectics) at 37°C, before and after completion of each study. Following calibration, the catheter was placed through the nostril into the esophagus. The exact position was determined by retraction of the probe after an acidic reading from the stomach had been obtained. Data were analyzed using Esophagram software (Medtronic Synectics). The percentage of time pH <4 (reflux index) was calculated. Pathological gastroesophageal reflux was defined as a reflux index >5% [14].

### 2.5. Immunological Study

Assay of serum samples for total immunoglobulin E (IgE) and fluorescent enzyme immunoassays (RAST-FX5) (Cap System, Pharmacia & Upjohn Diagnostics AB, Uppsala, Sweden) was performed for some children. RAST-FX5 is a mix test that can detect specific IgE to 6 major food allergens including cow's milk proteins, egg white, peanut, soy, wheat, and fish. Skin prick test was performed by one of the authors (AA) as follows: standard allergen extracts and Alyostal ST-IR (Stallergenes S.A. France) were used for the skin prick test. Antihistamines, H1, and H2 had to be withdrawn 14 days in advance. Allergen extracts were applied onto the skin of the ventral surface of the forearm after being wiped with alcohol. Histamine-HCl and NaCl were used as positive and negative controls, respectively. The results was evaluated 10–15 min later. An induration of >3 mm for positive control and <3 mm for negative control were accepted as a validity criterion for the test. A positive skin reaction was accepted if the reaction against the allergen resulted in an induration of >3 mm in diameter. Each test for individual consisted of 25 different foods extracts, 21 common inhalant extracts, and positive and negative controls.

### 2.6. Statistical Analysis

Statistical analysis was performed using Statistical Package for Social Sciences version 19 (SPSS, Inc, Chicago, IL, USA). Data were expressed as a percentage of the total for categorical variables, as a mean with standard deviation (SD) for normally distributed continuous variables, or as median with interquartile range for skewed distributed variables. Paired *t*-test was used to compare the mean eosinophil count before and after treatment. *P* value less than 0.05 was considered significant.

## 3. Results

### 3.1. Relative Frequency and Clinical Presentation

Out of the total of 2127 children who underwent upper endoscopy for upper gastrointestinal symptoms between 2002 and 2011, 312 (14.7%) children were diagnosed with esophagitis of various etiologies. Only 18 were diagnosed with EE constituting 5.8% of the cases of esophagitis and 0.85% of total number of patients who underwent upper endoscopy in the study period. The clinical and laboratory characteristics at presentation were shown in Table 1.

### 3.2. Extended Esophageal pH Study

Extended esophageal pH study performed on 4 patients showed reflux index of 2.1%, 3.5%, 2.9%, and 2.4%, respectively, indicating normal results.

### 3.3. Allergy Testing

Allergy testing and evaluation revealed that 7/18 (38.9%) had increased peripheral absolute eosinophil count (normal, 0.04–0.45 × 10^9^/L) and percentage (normal, 1–6%). High total IgE levels (normal, <60 IU/mL) were reported in 9/13 (69%) of the patients. The mean absolute eosinophil count, percentage, and IgE level were shown in Table 1. The most common food allergens tested positive in our patients by skin prick test were peanuts (*n* = 6), eggs (*n* = 6), hazelnuts (*n* = 4), wheat (*n* = 4), and sesame (*n* = 3). 

### 3.4. Endoscopic and Histopathological Findings

**Figure 1:**
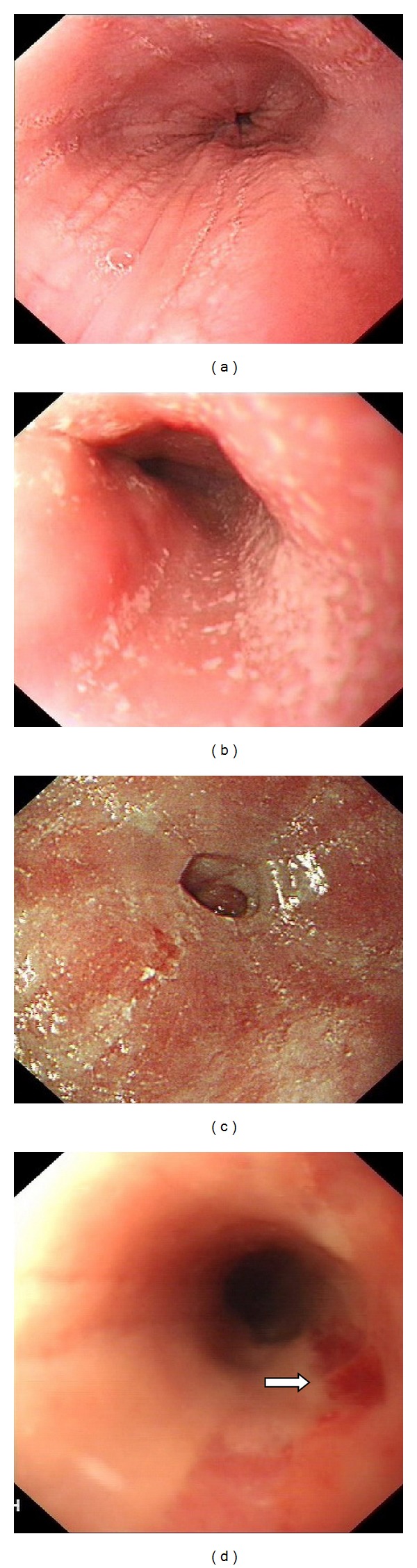
Endoscopic abnormalities seen in our patients. (a) Longitudinal furrows and edema giving the wrinkled appearance (patient 12). (b) Patchy specks or exudates mimicking esophageal candidiasis (patient 13). (c) Ring stricture at the lower esophagus (patient 14). (d) Crepe-paper mucosa in a narrow lumen esophagus (arrow) (patient 18).

The classical endoscopic abnormalities were demonstrated in Table 2. Presence of longitudinal furrow and loss of vascular pattern were the most common findings (Figure 1(a)), followed by patchy specks or exudates (Figure 1(b)), stricture (Figure 1(c)), Crepe paper (Figure 1(d)), and concentric rings.

**Figure 2:**
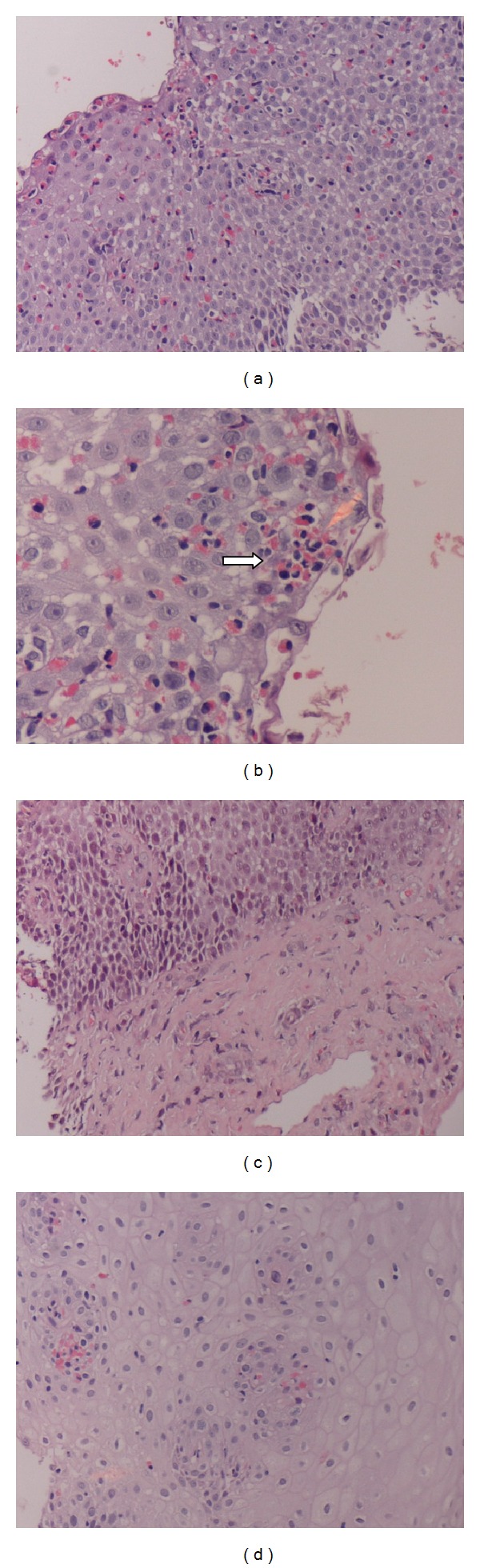
Histopathological abnormalities in our patients with EE. (a) Esophageal mucosa in a patient with eosinophilic esophagitis. Note the marked eosinophilic infiltration close to the surface (20X). (b) An aggregate of eosinophils forming eosinophilic microabscess (arrow) (40X). (c) Fibrosis of lamina propria and lamina propria eosinophils (20X). (d) Follow-up biopsy showing marked reduction in intraepithelial eosinophils and absence of eosinophilic microabscess, intercellular edema, and basal cell hyperplasia (20X).

The histopathological features of esophageal biopsies at initial diagnosis of eosinophilic esophagitis are summarized in Table 2. Markedly increased intraepithelial eosinophils were noticed in the majority of esophageal biopsies. The mean eosinophil count per hpf ± SD was 90.4 ± 33.6 (range, 28–152). The eosinophils were more dense in the surface layers (Figure 2(a)). Basal cell hyperplasia was noticed in all biopsies. Intercellular edema, eosinophilic degranulation, lamina propria papillae elongation, and eosinophilic microabscesses (Figure 2(b)) were noticed in the majority of biopsies. The lamina propria was lacking in six biopsies (33.3%) making it difficult to assess for lamina propria fibrosis and lamina propria eosinophils. The lamina propria showed fibrosis and eosinophilic infiltration in almost 90% of the remaining biopsies (Figure 2(c)). Review of accompanying gastric and duodenal biopsies did not show increased numbers of eosinophils. 

**Figure 3:**
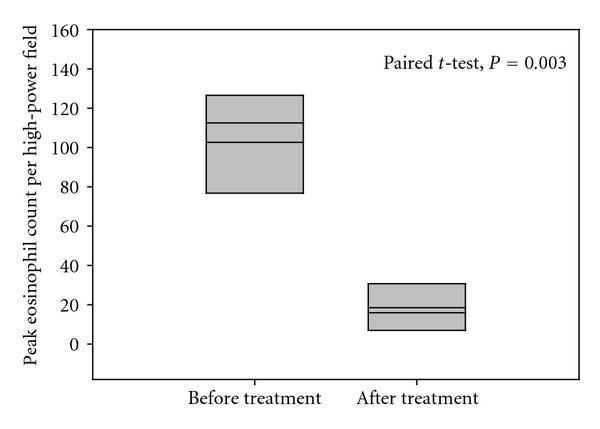
Changes in the mucosal mean peak eosinophil count following treatment in EE (*n* = 6).

Six patients underwent a second endoscopy and biopsy following treatment. In four patients, the eosinophilic count dropped significantly to counts less than 10/hpf (Figure 2(d)). Two patients showed moderate drop of eosinophilic count to >25/hpf. The changes in the peak eosinophilic count in histopathological examination were demonstrated in Figure 3. Interestingly, eosinophilic microabscesses disappeared in all follow-up biopsies. The other microscopic findings (intercellular edema, basal cell hyperplasia, lamina propria papillae elongation, and eosinophilic degranulation) decreased in some but not all follow-up biopsies.

### 3.5. Treatment and Followup

Six (33%) patients required treatment with oral prednisolone for 4 weeks (1–2 mg/kg/d, maximum 40 mg) followed by gradual weaning over 2 to 4 weeks and maintained with swallowed topical corticosteroids in small doses (50–100 *μ*g/day). The remaining 12 (66.7%) were treated with high dose of swallowed topical corticosteroids (500–1000 *μ*g/day) given twice daily in divided doses for 6 weeks followed by reduction to smaller dose for maintenance (50–100 *μ*g/day). The topical corticosteroids required were fluticasone in 14 patients, beclomethasone in 3, and budesonide in one patient. The patients were instructed to abstain from food and drinks and to rinse their mouth 30 minutes after swallowing the topical corticosteroids. None of our patients had oral Candida infections. Fifteen patients were started on proton pump inhibitor (PPI) before establishing the diagnosis of EE. Three of 5 patients with esophageal strictures required endoscopic balloon dilatation using (CRE Wireguided Balloon Dilator, Boston, S, C.). Dietary elimination was recommended for patients who had positive allergy testing either by skin prick test or by RAST-FX5 immunoassay.

The median duration for followup was 2.5 years (range, 0.5 to 10.1 years). All patients were advised to continue on swallowed low dose of topical corticosteroids. The followup was analyzed according to two subgroups: atopic patients with history of atopy and sensitization to foods (*n* = 10) and nonatopic patients (*n* = 8). Seven of the atopic patients and 3 of the nonatopic patients discontinued treatment on their own. Symptoms persisted in 4 of the atopic and 2 of the nonatopic patients. Two of the atopic patients who were not on treatment had an upper endoscopy after a period of followup of 9.5 and 10 years, respectively, that showed recurrence of their disease that was confirmed by histopathology.

## 4. Discussion

The prevalence of EE is not certain; however, a prevalence of 40–55 cases per 100,000 individuals has been estimated from western countries [4, 15]. For unknown reasons this disease predominantly affects males and individuals at all ages [1, 6, 16]. Males constituted 72.2% of our cohort in accordance with previous studies. In this paper, EE constituted 0.85% of the total number of children requiring upper endoscopy for upper gastrointestinal symptoms. This figure is comparable to the 1.18% reported out of 1700 patients seen at pediatric Gastroenterology Clinic at Oregon Health & Science University [17]. Interestingly the mean relative frequency of EE out of the total number of upper endoscopy performed in the first 3 years of our study was 0.29% as compared to 1.9% in the last 3 years of the study. Since the authors were fully aware about EE throughout the study period, this pattern reflects an actual increase rather than increased awareness of the condition in agreement with the study by Hruz and colleagues [4]. The 6-fold increase of frequency of EE observed in our study (from 2002–2005 to 2008–2011) may be related to the changes in food habits and consumptions.

The traditional meals in West Saudi Arabia consisted of rice, whole wheat, lamb, chicken, fish, fresh vegetables and dates, and other local fruits. Bread, eggs, cheese, beans, olive, and milk are usually served for breakfast and dinner. However, with the rapid expansion of US fast food chains over the last two decades, the food habits in the major cities in Saudi Arabia are becoming similar to the western's habits with increasing consumption of fast foods, frozen, and prepared foods in supermarkets and restaurants. There was also a noticeable increase in the use of peanut butter. Eating at fast food outlets was found to be a significant risk factor for bronchial asthma development in a study of children from the city of Jeddah and Saudi villages where the traditional dietary habits persisted [18]. 

In general, although there were no published data about the prevalence of food allergy in Saudi Arabia, the prevalence of other allergic disorders such as bronchial asthma and allergic rhinitis was increasing [19, 20]. The prevalence of EE has been considered the lowest among other allergic diseases [21]. Our study constituted only of children of Arab ethnicity. More than half of our patients had history of one or more allergic diseases and half of them had positive first-degree relatives with allergic diseases. Children with EE often have other associated allergic diseases such as allergic rhinitis, bronchial asthma, atopic dermatitis, and food allergy [8–10]. Food allergy in children has been linked to EE [22]. In this study, evidence for food allergy was obtained by testing for food-specific IgE antibodies in the serum in 10 patients and by skin prick testing in 11 patients. Testing for food-specific antibodies in the serum has low negative predictive value as negative test was reported in association with allergic reaction in 10% to 25% of patients [23]. At the contrary, skin prick test possesses a high negative predictive value of 95% to 100% which makes it appropriate for exclusion of IgE-mediated food allergies of the 4 common allergenic foods: milk, egg, peanut, and fish [23].

The implication of testing for IgE-mediated food allergy in the management of EE was studied by many authors. Liacouras and colleagues [8] reported improvement in 57% of children with EE with dietary restriction guided by results of skin prick and patch testing. Elimination of the most common six food items including milk, wheat, soy, egg, peanut, and seafood was reported to result in improvement of symptoms and histology in children with EE [24]. Many authors [8, 24, 25] reported benefit using elemental diet. In a recent retrospective study of 98 children with EE, when elemental diet, selected 6-food elimination, and skin prick test-guided elimination were compared, remission rate of 96%, 81%, and 65% was achieved [26]. 

In our patients with EE we practiced simple food elimination guided by history and positive skin prick testing, none of our patients had received elemental diet or extensive food elimination, as we found low compliance because of palatability, cost, and availability. In addition, considering the chronicity of the disease and the tendency to recur after discontinuation of treatment, lifelong food elimination may be difficult [27]. The promising solution may rely on finding specifically the offending allergen as reported recently by Gonsalves and colleagues [28] in adults with EE during systematic reintroduction of food items following 6-food elimination diet for 6 weeks. This approach may help in ameliorating the disease process in children with EE.

Increased number of intraepithelial eosinophils has been regarded as the key diagnostic criterion for diagnosis of EE. In this study we choose the presence of intraepithelial eosinophils >15 in >2 hpfs or >25 in any single hpf as our definition of EE to limit the possibility of including children with gastroesophageal reflux. All patients included fulfilled this criterion. Additionally, clusters of eosinophils forming microabscess were found in 77.8% of our patients. The presence of eosinophilic microabscesses is strongly supportive of the diagnosis of EE but not GERD [13, 29]. Interestingly, those microabscesses disappeared in follow-up biopsies after treatment. Furthermore, eosinophil degranulation that was seen in the majority of biopsies obtained from our patients further supports the diagnosis of EE as opposed to GERD [13, 29]. Only 3 studies [13, 30, 31] reported subepithelial fibrosis of the lamina propria in EE because lamina propria is usually absent in most esophageal pinch biopsy specimens. In our study, 12 biopsy specimens contained lamina propria and were adequate for evaluation for fibrosis in which lamina propria fibrosis was seen in 91.7% of the specimens studied. The lamina propria fibrosis has been linked to the presence of dysphagia in one study [30]. 

There is still uncertainty about the optimal treatment given to patients with EE and the impact of treatment on the long-term outcome of the disease. In our series of children with EE, 6 patients were treated with systemic corticosteroids in the form of oral prednisolone. This approach was supported by an earlier study by Liacouras and colleagues [32] who reported improvement of 20 children with EE out of 21 treated with oral methylprednisolone. A course of systemic steroid may be considered for patients with severe dysphagia resulting in significant weight loss or esophageal stricture at risk for perforation before attempted dilatation.

Swallowed topical steroids either beclomethasone or fluticasone propionate have been reported to be effective in improving symptoms and histology in the majority of treated children with EE [2, 8]. In our cohort, all patients required topical corticosteroids. In 12 patients disease remission was achieved by high dose of topical corticosteroids only followed by low dose for maintenance. Compliance with maintenance treatment was very poor as 10 patients discontinued the treatment at some time during followup. Since the disease tended to be chronic with tendency to recur after discontinuation of treatment this approach of low-dose maintenance needs to be further evaluated in a larger scale of studies. 

This study is limited by its retrospective nature, the relatively small number of patients, and the lack of standardization of the treatment protocol. Future studies should evaluate the need for maintenance treatment, the role for dietary elimination and reintroduction in finding the offending food allergens, the natural course of the disease, and the long-term complications.

In conclusion, eosinophilic esophagitis is an emerging disease that needs to be considered in any child presenting with esophageal dysfunction, unexplained stricture, or gastroesophageal reflux symptoms unresponsive to medical treatment of reflux.

## Figures and Tables

**Table 1:** Clinical and laboratory characteristics of children with eosinophilic esophagitis (*n* = 18).

Median age, years (range)	8.5 (1.5–18)
Males, *n* (%)	13 (72.2)
Symptoms, *n* (%)	
Dysphagia	11 (61.1)
Vomiting	10 (55.6)
Food impaction	4 (22.2)
Feeding aversion	3 (16.7)
Poor weight gain	2 (11.1)
History of atopy, *n* (%)	10 (55.6)
First degree relatives with atopy, *n* (%)	9 (50)
Growth parameters	
Weight for age *z*-score, mean ± SD (range)	0.59 ± 1.5 (−2.3–2.9)
Height for age *z*-score, mean ± SD (range)	0.13 ± 1.3 (−2.1–2.1)
Allergic testing and evaluation	
Absolute eosinophil counts, mean ± SD (range)	0.48 ± 0.44 (0.05–1.8)
Eosinophil percentage, mean ± SD (range)	5.5 ± 3.8 (1–12.2)
IgE level (IU/mL), mean ± SD (range)	259.9 ± 530.4 (26–1848)
Positive RAST-FX5, *n* (%)	10 (55.6)
Positive skin prick test for food allergens, *n* (%)	11 (61.1)
Positive skin prick test for aeroallergens, *n* (%)	3 (16.7)

**Table 2:** Endoscopic and histopathological characteristics of children with EE.

	Number/total	(%)
Endoscopic findings		
Longitudinal furrows	17/18	(94.4)
Loss of vascular pattern	17/18	(94.4)
Patchy whitish exudates	7/18	(38.9)
Stricture	5/18	(27.8)
Concentric mucosal rings	2/18	(11)
Crepe paper	2/18	(11)
Normal mucosa	1/18	(5.6)
Histopathological features		
Eosinophilic microabscesses	14/18	(77.8)
Intercellular edema	17/18	(94.4)
Basal cell hyperplasia	18/18	(100)
Lamina propria papillae elongation	14/18	(77.8)
Lamina propria fibrosis	11/12	(91.7)
Lamina propria eosinophils	11/12	(91.7)
Eosinophilic degranulation	17/18	(94.4)
Neutrophil infiltration	2/18	(11.1)
Ulceration	1/18	(5.6)

## References

[1] Liacouras CA, Furuta GT, Hirano I (2011). Eosinophilic esophagitis: updated consensus recommendations for children and adults. *Journal of Allergy and Clinical Immunology*.

[2] Noel RJ, Putnam PE, Rothenberg ME (2004). Eosinophilic esophagitis. *The New England Journal of Medicine*.

[3] Straumann A, Simon HU (2005). Eosinophilic esophagitis: escalating epidemiology?. *Journal of Allergy and Clinical Immunology*.

[4] Hruz P, Straumann A, Bussmann C (2011). Escalating incidence of eosinophilic esophagitis: a 20-year prospective, population-based study in Olten County, Switzerland. *Journal of Allergy and Clinical Immunology*.

[5] Franciosi JP, Liacouras CA (2009). Eosinophilic esophagitis. *Immunology and Allergy Clinics of North America*.

[6] Furuta GT, Liacouras CA, Collins MH (2007). Eosinophilic esophagitis in children and adults: a systematic review and consensus recommendations for diagnosis and treatment. *Gastroenterology*.

[7] Gonsalves N (2008). Eosinophilic esophagitis: history, nomenclature, and diagnostic guidelines. *Gastrointestinal Endoscopy Clinics of North America*.

[8] Liacouras CA, Spergel JM, Ruchelli E (2005). Eosinophilic esophagitis: a 10-year experience in 381 children. *Clinical Gastroenterology and Hepatology*.

[9] Orenstein SR, Shalaby TM, Di Lorenzo C, Putnam PE, Sigurdsson L, Kocoshis SA (2000). The spectrum of pediatric eosinophilic esophagitis beyond infancy: a clinical series of 30 children. *American Journal of Gastroenterology*.

[10] Simon D, Marti H, Heer P, Simon HU, Braathen LR, Straumann A (2005). Eosinophilic esophagitis is frequently associated with IgE-mediated allergic airway diseases. *Journal of Allergy and Clinical Immunology*.

[11] Hasosah MY, Sukkar GA, Alsahafi AF (2011). Eosinophilic esophagitis in Saudi children: symptoms, histology and endoscopy results. *Saudi Journal of Gastroenterology*.

[12] Al-Hussaini A, Semaan T, El Hag I (2009). Esophageal trachealization: a feature of eosinophilic esophagitis. *Saudi Journal of Gastroenterology*.

[13] Parfitt JR, Gregor JC, Suskin NG, Jawa HA, Driman DK (2006). Eosinophilic esophagitis in adults: distinguishing features from gastroesophageal reflux disease: a study of 41 patients. *Modern Pathology*.

[14] Schindlbeck NE, Heinrich C, Konig A (1987). Optimal thresholds, sensitivity, and specificity of long-term pH-metry for the detection of gastroesophageal reflux disease. *Gastroenterology*.

[15] Prasad GA, Alexander JA, Schleck CD (2009). Epidemiology of eosinophilic esophagitis over three decades in Olmsted County, Minnesota. *Clinical Gastroenterology and Hepatology*.

[16] Kapel RC, Miller JK, Torres C, Aksoy S, Lash R, Katzka DA (2008). Eosinophilic esophagitis: a prevalent disease in the United States that affects all age groups. *Gastroenterology*.

[17] Eroglu Y, Lu H, Terry A (2009). Pediatric eosinophilic esophagitis: single-center experience in northwestern USA. *Pediatrics International*.

[18] Hijazi N, Abalkhail B, Seaton A (2000). Diet and childhood asthma in a society in transition: a study in urban and rural Saudi Arabia. *Thorax*.

[19] Al Frayh AR, Shakoor Z, Gad El Rab MO, Hasnain SM (2001). Increased prevalence of asthma in Saudi Arabia. *Annals of Allergy, Asthma and Immunology*.

[20] Nahhas M, Bhopal R, Anandan C, Elton R, Sheikh A (2012). Prevalence of allergic disorders among primary school-aged children in Madinah, Saudi Arabia: two-stage cross-sectional survey. *PLoS ONE*.

[21] Hopp RJ (2012). Eosinophilic esophagitis in pediatrics: the worst of all possible allergy worlds?. *Journal of Allergy*.

[22] Hong S, Vogel NM (2010). Food allergy and eosinophilic esophagitis: learning what to avoid. *Cleveland Clinic Journal of Medicine*.

[23] Sampson HA (2001). Utility of food-specific IgE concentrations in predicting symptomatic food allergy. *Journal of Allergy and Clinical Immunology*.

[24] Kagalwalla AF, Sentongo TA, Ritz S (2006). Effect of six-food elimination diet on clinical and histologic outcomes in eosinophilic esophagitis. *Clinical Gastroenterology and Hepatology*.

[25] Markowitz JE, Spergel JM, Ruchelli E, Liacouras CA (2003). Elemental diet is an effective treatment for eosinophilic esophagitis in children and adolescents. *American Journal of Gastroenterology*.

[26] Henderson CJ, Abonia JP, King EC (2012). Comparative dietary therapy effectiveness in remission of pediatric eosinophilic esophagitis. *Journal of Allergy and Clinical Immunology*.

[27] Debrosse CW, Franciosi JP, King EC (2011). Long-term outcomes in pediatric-onset esophageal eosinophilia. *Journal of Allergy and Clinical Immunology*.

[28] Gonsalves N, Yang G-Y, Doerfler B, Ritz S, Ditto AM, Hirano I (2012). Elimination diet effectively treats eosinophilic esophagitis in adults; Food reintroduction identifies causative factors. *Gastroenterology*.

[29] Desai TK, Stecevic V, Chang CH, Goldstein NS, Badizadegan K, Furuta GT (2005). Association of eosinophilic inflammation with esophageal food impaction in adults. *Gastrointestinal Endoscopy*.

[30] Chehade M, Sampson HA, Morotti RA, Magid MS (2007). Esophageal subepithelial fibrosis in children with eosinophilic esophagitis. *Journal of Pediatric Gastroenterology and Nutrition*.

[31] Straumann A, Rossi L, Simon HU, Heer P, Spichtin HP, Beglinger C (2003). Fragility of the esophageal mucosa: a pathognomonic endoscopic sign of primary eosinophilic esophagitis?. *Gastrointestinal Endoscopy*.

[32] Liacouras CA, Wenner WJ, Brown K, Ruchelli E (1998). Primary eosinophilic esophagitis in children: successful treatment with oral corticosteroids. *Journal of Pediatric Gastroenterology and Nutrition*.

